# Correction to: Minimum Quality Threshold in Pre-Clinical Sepsis Studies (MQTiPSS): an international expert consensus initiative for improvement of animal modeling in sepsis

**DOI:** 10.1007/s15010-018-1197-2

**Published:** 2018-09-17

**Authors:** Marcin F. Osuchowski, Alfred Ayala, Soheyl Bahrami, Michael Bauer, Mihaly Boros, Jean-Marc Cavaillon, Irshad H. Chaudry, Craig M. Coopersmith, Clifford Deutschman, Susanne Drechsler, Philip Efron, Claes Frostell, Gerhard Fritsch, Waldemar Gozdzik, Judith Hellman, Markus Huber-Lang, Shigeaki Inoue, Sylvia Knapp, Andrey V. Kozlov, Claude Libert, John C. Marshall, Lyle L. Moldawer, Peter Radermacher, Heinz Redl, Daniel G. Remick, Mervyn Singer, Christoph Thiemermann, Ping Wang, W. Joost Wiersinga, Xianzhong Xiao, Basilia Zingarelli

**Affiliations:** 1 Ludwig Boltzmann Institute for Experimental and Clinical Traumatology in the AUVA Research Center, Donaueschingenstrasse 13, 1200 Vienna, Austria; 2 Rhode Island Hospital and Alpert School of Medicine at Brown University, Providence, RI USA; 3 Jena University Hospital, Jena, Germany; 4 Institute of Surgical Research, University of Szeged, Szeged, Hungary; 5 Institut Pasteur, Paris, France; 6 University of Alabama at Birmingham School of Medicine, Birmingham, AL USA; 7 Emory University School of Medicine, Atlanta, GA USA; 8 Feinstein Institute for Medical Research, Northwell Health, Manhasset, NY USA; 9 University of Florida College of Medicine, Gainesville, FL USA; 10 Division of Anaesthesia and Intensive Care, Karolinska Institutet, Danderyd Hospital, Stockholm, Sweden; 11 AUVA Traumacenter, Vienna, Austria; 12 Paracelsus Medical University, Salzburg, Austria; 13 Wroclaw Medical University, Wroclaw, Poland; 14 School of Medicine, University of California, San Francisco, San Francisco, CA USA; 15 Institute of Clinical and Experimental Trauma-Immunology, University Hospital of Ulm, Ulm, Germany; 16 Kobe University Graduate School of Medicine, Kobe, Japan; 17 Department of Medicine 1, Medical University Vienna, Vienna, Austria; 18 Center for Inflammation Research, VIB, Ghent, Belgium; 19 University Ghent, Ghent, Belgium; 20 Keenan Research Centre for Biomedical Science, St. Michael’s Hospital, University of Toronto, Toronto, Canada; 21 Institute of Anaesthesiological Pathophysiology and Process Development, University Hospital of Ulm, Ulm, Germany; 22 Boston University School of Medicine, Boston, MA USA; 23 Bloomsbury Institute of Intensive Care Medicine, University College London, London, UK; 24 The William Harvey Research Institute, Barts and London School of Medicine and Dentistry, Queen Mary University of London, London, UK; 25 Feinstein Institute for Medical Research, Manhasset, NY USA; 26 Division of Infectious Diseases, and Center for Experimental and Molecular Medicine, the Academic Medical Center, University of Amsterdam, Amsterdam, The Netherlands; 27 Xiangya School of Medicine, Central South University, Chagnsha, Hunan China; 28 Division of Critical Care Medicine, Cincinnati Children’s Hospital Medical Center, College of Medicine, University of Cincinnati, Cincinnati, OH USA

## Correction to: Infection 10.1007/s15010-018-1183-8

The original version of this article unfortunately contained mistakes.

The Tables 1–3 were missing. The correct versions of Tables 1, 2 and 3 are given below.

**Table 1:** Combined Recommendations and Considerations from the Working Group (WG) 1 and 2

**Study Design** (WG-1)	**1.** Survival follow-up should reasonably reflect the clinical time course of the sepsis model	**R**
	**2.** Therapeutic interventions should be initiated after the septic insult replicating clinical care	
	**3.** We recommend that the treatment be randomized and blinded when feasible	
	**4.** Provide as much information as possible (e.g. ARRIVE guidelines) on the model and methodology, to enable replication.	
	*a. Consider replication of the findings in models that include co-morbidity and/or other biological variables (i.e., age, gender, diabetes, cancer, immuno-suppression, genetic background and others).*	**C**
	*b. In addition to rodents (mice and rats), consider modeling sepsis also in other (mammal) species.*	
	*c. Consider need for source control*	
**Humane Modeling** (WG-2)	**5.** The development and validation of standardized criteria to monitor the well-being of septic animals is recommended	**R**
	**6.** The development and validation of standardized criteria for euthanasia of septic animals is recommended (exceptions possible)	
	**7.** Analgesics recommended for surgical sepsis consistent with ethical considerations	
	*d. Consider analgesics for nonsurgical sepsis*	**C**

R: Recommendation strength; C: consideration strength

**Table 2:** Combined Recommendations and Considerations from the Working Group (WG) 3 and 4

**Infection Types** (WG-3)	**8.** We recommend that challenge with LPS is not an appropriate model for replicating human sepsis	**R**
	**9.** We recommend that microorganisms used in animal models preferentially replicate those commonly found in human sepsis	
	*e. Consider modeling sepsis syndromes that are initiated at sites other than the peritoneal cavity (e.g. lung, urinary tract, brain)*	**C**
**Organ Failure/ Dysfunction** (WG-4)	**10.** Organ/system dysfunction is defined as life threatening deviation from normal for that organ/system based on objective evidence	**R**
	**11.** Not all activities in an individual organ/system need to be abnormal for organ dysfunction to be present	
	**12.** To define objective evidence of the severity of organ/system dysfunction, a scoring system should be developed, validated and used, or use an existing scoring system.	
	**13.** Not all experiments must measure all parameters of organ dysfunction but animal models should be fully exploited	
	*f. Avoid hypoglycemia*	**C**

R: Recommendation strength; C: consideration strength

**Table 3:** Combined Recommendations and Considerations from the Working Group (WG) 5 and 6

**Fluid Resuscitation** (WG-5)	**14.** Fluid resuscitation is essential unless part of the study	**R**
	**15.** Administer fluid resuscitation based on the specific requirements of the model	
	**16.** Consider the specific sepsis model for the timing of the start and continuation for fluid resuscitation	
	**17.** Resuscitation is recommended by the application of iso-osmolar crystalloid solutions	
	*g. Consider using pre-defined endpoints for fluid resuscitation as deemed necessary*	**C**
	*h. Avoid fluid overload*	
**Anti-microbial Therapy** (WG-6)	**18.** Antimicrobials are recommended for pre-clinical studies assessing potential human therapeutics	**R**
	**19.** Antimicrobials should be chosen based on the model and likely/known pathogen	
	**20.** Administration of antimicrobials should mimic clinical practice	
	*i. Antimicrobials should be initiated after sepsis is established*	**C**

R: Recommendation strength; C: consideration strength

Bettina Standhartinger was unfortunately not correctly named in the acknowledgments of the original version of this article. The correct acknowledgements are as follows:

 The authors would like to thank Bettina Standhartinger for her valuable assistance in organizing the Wiggers–Bernard Conference.

The original article has been corrected.

